# Congenitally corrected transposition of the great arteries: one disease, diverse manifestations—a case series

**DOI:** 10.1093/ehjcr/ytaf174

**Published:** 2025-04-08

**Authors:** Maria Luisa Benesch Vidal, Han Ki Park, Kouichi Toda, Geetha Kandavello, Lucy Youngmin Eun, Dominica Zentner, Motoki Komori, Christoph Sinning

**Affiliations:** Department of Cardiology, University Heart and Vascular Centre Hamburg, Martinistr. 52, 20251 Hamburg, Germany; Division of Cardiovascular Surgery, Department of Thoracic and Cardiovascular Surgery, Severance Cardiovascular Hospital, Yonsei University College of Medicine, 50-1, Yonsei-Ro, Seodaemun-gu, Seoul 03722, Korea; Department of Cardiovascular Surgery, Dokkyo Medical University Saitama Medical Centre, 2 Chome-1-50 Minamikoshigaya, Koshigaya, Saitama 343-8555, Japan; Paediatric and Congenital Heart Centre, Institute Jantung Negara (National Heart Institute), 145, Jln Tun Razak, 50400 Kuala Lumpur, Wilayah Persekutuan Kuala Lumpur, Malaysia; Division of Paediatric Cardiology, Department of Paediatrics, Yonsei University College of Medicine, 50-1 Yonsei-ro, Seodaemun-gu, Seoul, Korea; Department of Cardiology, The Royal Melbourne Hospital, Walter and Eliza Hall Institute/1G Royal Parade, Parkville, Melbourne, VIC 3050, Australia; Faculty of Medicine, Dentistry and Health Sciences, Royal Melbourne Hospital Clinical School, University of Melbourne, 20 Flemington Rd, Parkville, Melbourne, VIC 3050, Australia; Department of Paediatric Cardiovascular Surgery, National Cerebral and Cardiovascular Centre, 6-1 Kishibeshinmachi, Suita, Osaka 564-8565, Japan; Department of Cardiology, University Heart and Vascular Centre Hamburg, Martinistr. 52, 20251 Hamburg, Germany

**Keywords:** Congenitally corrected transposition of the great arteries, Heart failure, Ventricular assist device, Cardio-obstetric management, Coronary artery disease, Case series

## Abstract

**Background:**

Congenitally corrected transposition of the great arteries (ccTGA) is a rare congenital heart defect with heterogenous clinical manifestations that can pose both diagnostic and management challenges throughout life.

**Case summary:**

We describe four patients with ccTGA and different presentations including sudden cardiac arrest, progressive heart failure, post-partum heart failure, and NSTEMI.

**Aims and Discussion:**

This case series aims to illustrate the importance of multimodality imaging to assist the diagnosis and support treatment strategies in patients with ccTGA. Direct long-term sequalae, such as arrythmias and heart failure, and the associated management challenges are highlighted. Additionally, the challenges of managing pregnancy with a sRV and the development of acquired heart disease demonstrate the clinical care challenges in caring for this population across the life span.

**Learning points**

- Patients affected by ccTGA with balanced hemodynamics can remain asymptomatic into adulthood. Symptoms are mostly related to arrhythmias, AV conduction abnormalities, worsening sRV function and tricuspid valve insufficiency.
- Multimodality imaging, CPET, RHC and serial assessment of cardiac biomarkers are essential to diagnosis and management in ccTGA.
- ccTGA patients should be referred to an adult with congenital (ACHD) expert centre at diagnosis for surveillance of long-term sequalae and complications, so early initiation of appropriate interventions can be initiated.
- End-stage heart failure in ccTGA poses major challenges and ventricular assist device implantation can function as a bridge to transplant or destination therapy.
- Pregnancy in ccTGA patients requires pre-conception counselling. Regular review at a specialized ACHD centre with a cardio-obstetric unit is recommended.
- Acquired heart disease such as coronary artery diseases, can affect ccTGA patients with variant anatomy of the coronary arteries as first indicative findings.

## Introduction

Congenitally corrected transposition of the great arteries (ccTGA) is a rare congenital heart defect (CHD), accounting for ∼0.5% of all CHD.^1–3^ ccTGA is characterized by atrio-ventricular and ventriculo-arterial discordance. It is often accompanied by associated heart lesions, such as ventricular septal defect (VSD), pulmonary valve stenosis (PS) or atrial septal defect. With advances in pre-natal assessment and diagnosis, physiologic surgical repair has increased throughout the last decades.^1,4^ Nevertheless, particularly in ccTGA without accompanying defects, symptoms can be mild and clinical signs absent. Diagnosis is often delayed,^2^ precipitated by the development of arrhythmias or heart failure symptoms due to worsening systemic right ventricular (sRV) function and progressive systemic AV valve regurgitation.^1–3^ The varied anatomy and haemodynamic sequalae can lead to diagnostic challenges for physicians and additionally a patient cohort with little evidence-based guidance for management.^1–3^ This case series presents complete follow-up of four patients with ccTGA, highlighting the variability in clinical presentations, timing of diagnosis, and the management approaches guided by current evidence, guidelines, and expert consensus.

## Summary figure

**Figure:**
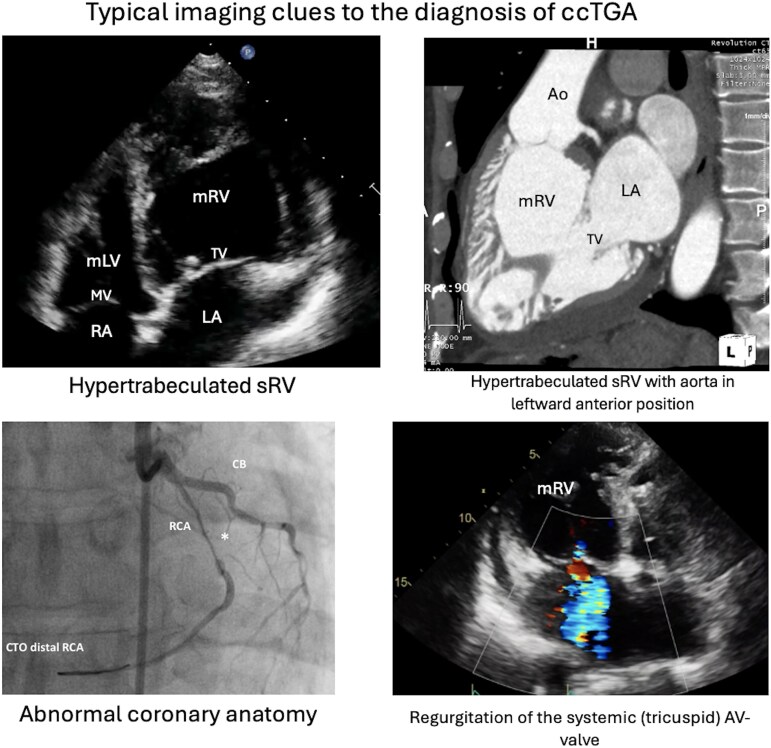


## Case 1: arrhythmia and sudden cardiac arrest in undiagnosed ccTGA with impaired ventricular function

**Figure 1:**
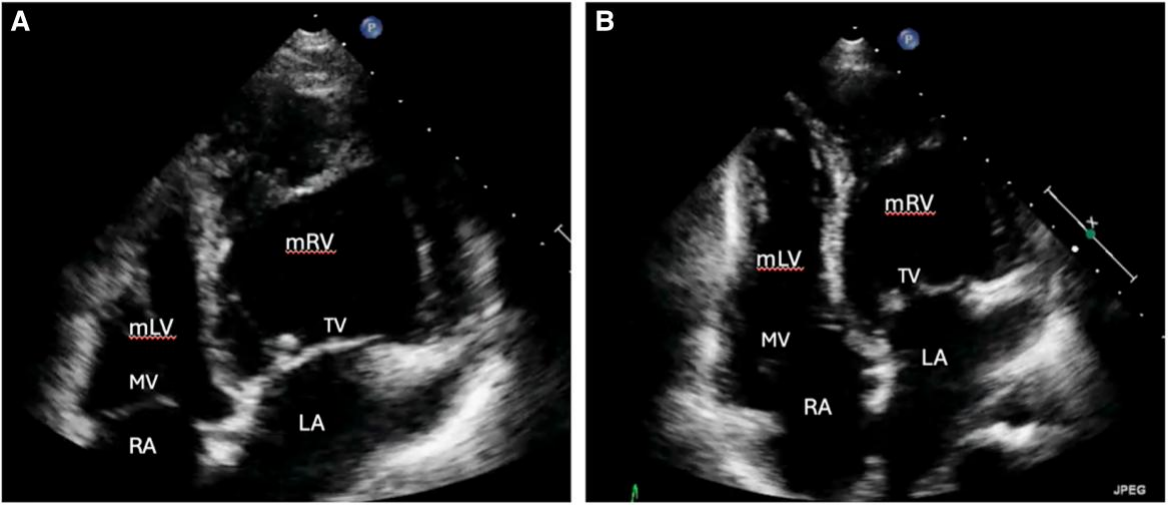
Transthoracic echocardiographic findings of a 42-year-old woman after OHCA. The right ventricle shows predominant apical trabeculation and the presence of a moderator band *(A)*. Additionally, the apically displaced tricuspid AV valve is displayed *(B)*. LA, left atrium; mLV, morphologic left ventricle; mRV, morphologic right ventricle; MV, mitral valve; RA, right atrium; TV, tricuspid systemic AV valve.

A 42-year-old woman presented to the emergency department following a resuscitated out-of-hospital cardiac arrest while driving. A CT scan excluded both pulmonary embolism and acute arterial dissection. Echocardiography demonstrated abnormal ventricular wall motion. Subsequent coronary angiography was normal. Further echocardiographic assessment revealed the right ventricle in the systemic position with mildly impaired ventricular function (*Figure 1*) with mild aortic and systemic tricuspid atrioventricular valve regurgitation. There were no other associated cardiac lesions. A cardiac CT-scan confirmed the diagnosis of ccTGA with the aorta in a leftward anterior position. A 24 h-holter revealed no arrhythmia. Insertion of a secondary prevention ICD was undertaken. A beta-blocker and an ACE-inhibitor were prescribed in the setting of mild sRV impairment, and the patient was discharged home. The patient has remained stable without any functional limitations. Regular ICD-interrogations have subsequently documented two brief episodes of atrial fibrillation (max. 52 s) and no ventricular arrhythmias. No anti-tachycardia therapy or shock has been delivered, representing a balanced status upon most recent clinical examination.

## Case 2: ccTGA and pregnancy—a multi-disciplinary team management approach

A 39-year-old female (G5P3M1) with previously uneventful pregnancies presented to the hospital with acute heart failure in the setting of mild hypertension 7 days post vaginal delivery of twins. Initial symptoms consisted of orthopnoea as well as shortness of breath during exertion, with further deterioration to breathlessness at rest 24 h preceding presentation. She was previously diagnosed to have ccTGA, restrictive VSD and subpulmonary stenosis.

The course of the pregnancy had been overall uncomplicated with respect to the CHD. Maternal cardiac care during pregnancy had consisted of 4 reviews in a multi-disciplinary cardio-obstetrics clinic, at gestations of 13, 24, 31, and 35 weeks. Cardiac investigations included repeated echocardiography, and 6-min walk tests with a single brain natriuretic peptide (BNP) at 24 weeks gestation.

Two years prior to conception, the patient had been stable, in sinus rhythm, and with a normal peak VO2max on CPET test. Pre-conception investigations included echocardiography a year prior, showing mild sRV systolic dysfunction and mild to moderate TR. A cardiac magnetic resonance (CMR) 5 months before, had quantified the sRV as having an end-diastolic volume index of 118 mL/m^2^ and an EF of 56%. Conception was planned, after these tests and discussion, and included withdrawal of an angiotensin converting enzyme inhibitor (ACEI).

Investigations at post-partum presentation revealed a BNP of 715 ng/L (normal < 100 ng/L), which was elevated compared with the mid-pregnancy result of 62 ng/L and a hsTn of 96 ng/L (normal < 16 ng/L). Chest x-ray initially revealed no signs of congestion. Computed tomography pulmonary angiogram, done mainly to exclude pulmonary embolism, showed bilateral ground glass changes and pleural fluid consistent with oedema, though both infection and inflammation were not able to be excluded. Full blood count was normal, and the patient was afebrile, however. Electrocardiogram remained unchanged in sinus rhythm.

A diagnosis of symptomatic heart failure was established, and intravenous (IV) diuretic treatment was instituted and delivered through a ‘Hospital in the Home’ program to facilitate breastfeeding and care of the patient’s newborn twins. She responded well to treatment, and her HF symptoms improved. Her diuretic was changed to oral, and an ACEi (enalapril, given breastfeeding status) was initiated.

At review a month later, the patient reported being well (NYHA FC I). An Implanon contraceptive rod had been inserted in the interim to prevent future pregnancy.

At 3 months post-partum, an echocardiogram reported a moderately dilated sRV with mildly reduced function. Mild to moderate TR was present with moderate pulmonary stenosis (mean gradient 29 mmHg). 6-min walk test distance was 490 m, with no desaturation. The patient has remained in stable condition upon most recent follow-up.

## Case 3: worsening systemic RV function and HF in ccTGA—device treatment and evaluation for ventricular assist device

A 37-year-old male presented to a regional medical centre with abdominal pain. A diagnosis of complete heart block was made during routine preoperative evaluation for acute appendicitis. Further cardiac workup revealed the diagnosis of ccTGA, with no other associated lesion. A permanent dual-chamber pacemaker was implanted

During follow-up, he gradually developed exertional dyspnoea and diuretic therapy was started. At the age of 45, worsening exertional dyspnoea and palpitations, lead to a diagnosis of atrial fibrillation. Cardioversion was attempted but was unsuccessful, and medical therapy was expanded to include ACE-inhibitor, a beta-blocker, and oral anticoagulation.

**Figure 2:**
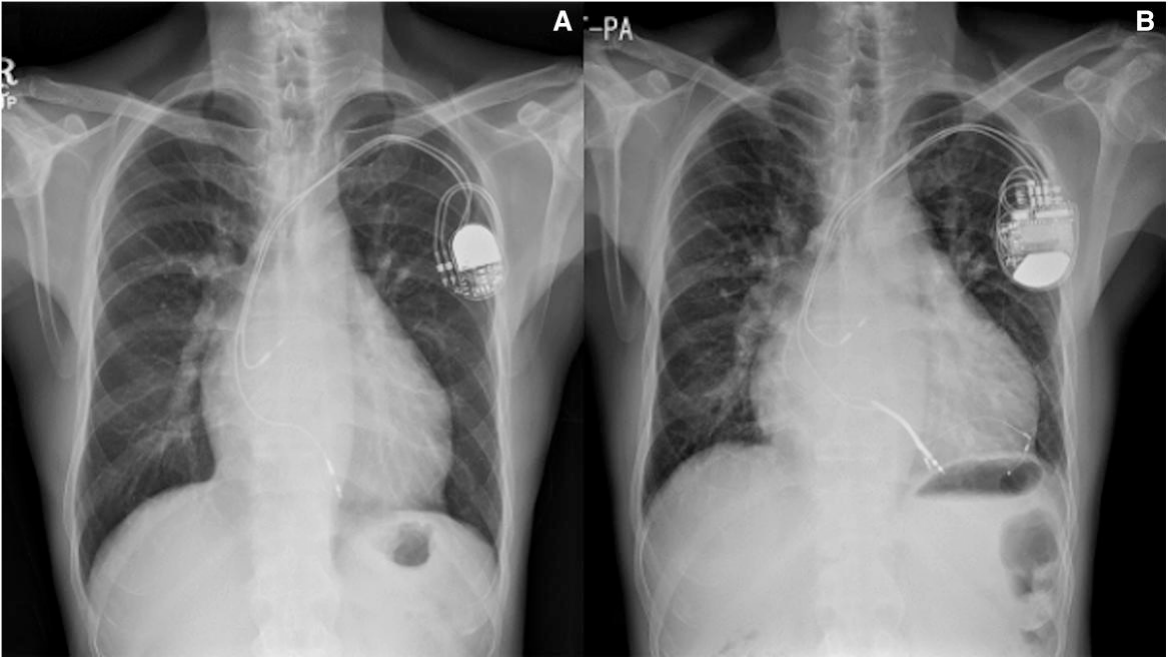
Chest radiography of a 57-year-old male patient with ccTGA, post-implantation of a dual-chamber PPM for complete heart block with mild cardiomegaly *(A)* and worsening sRV failure over time *(B)* despite upgrade to CRT.

By the age of 57, heart failure symptoms had progressed to NYHA FC III, prompting referral from his general cardiologist to a specialized adult with congenital heart disease (ACHD) centre. Chest radiography revealed mild cardiomegaly (*Figure 2A*).

**Figure 3:**
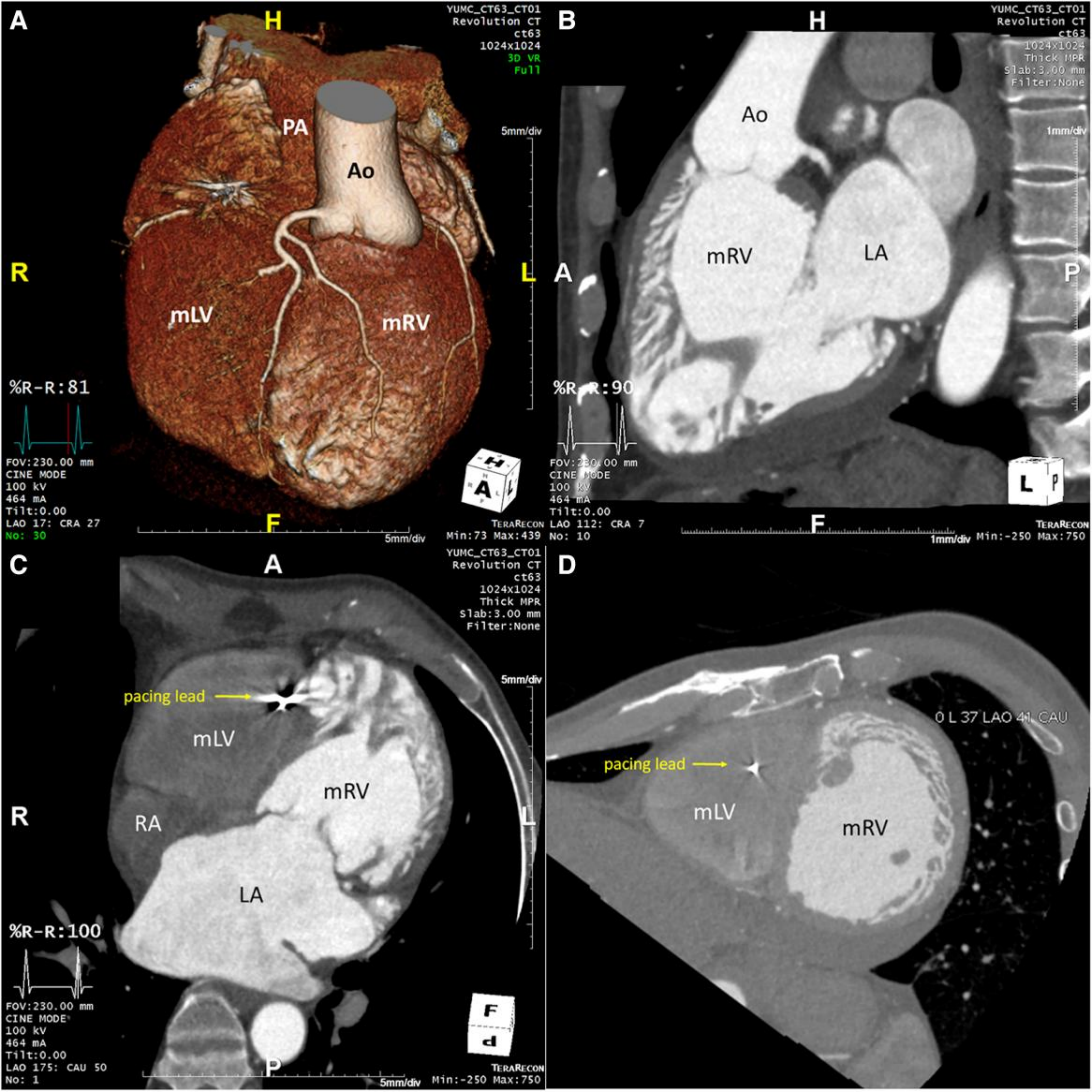
Cardiac CT of a 57-year old male patient confirming the diagnosis of ccTGA. *(A)*: 3D-anatomical reconstruction revealing L-transposition of the great arteries with the aorta in left anterior position arising from the systemic morphologic right ventricle. *(B)*: CT 3-chamber view revealing progressive RA- and RV-dilation with a sRV in sub-aortic position and a tricuspid atrioventricular valve. *(C)*: CT 4-chamber view revealing RA- and RV-dilation as well as the pacemaker-wire in the apical subpulmonary LV. *(D)*: CT short axis view at the papillary muscle level revealing the left sided and dilated systemic RV in leftward position. Ao, Aorta; LA, left atrium; mLV, morphologic left ventricle; mRV, morphologic right ventricle; PA, pulmonary artery; RA, right atrium.

**Figure 4:**
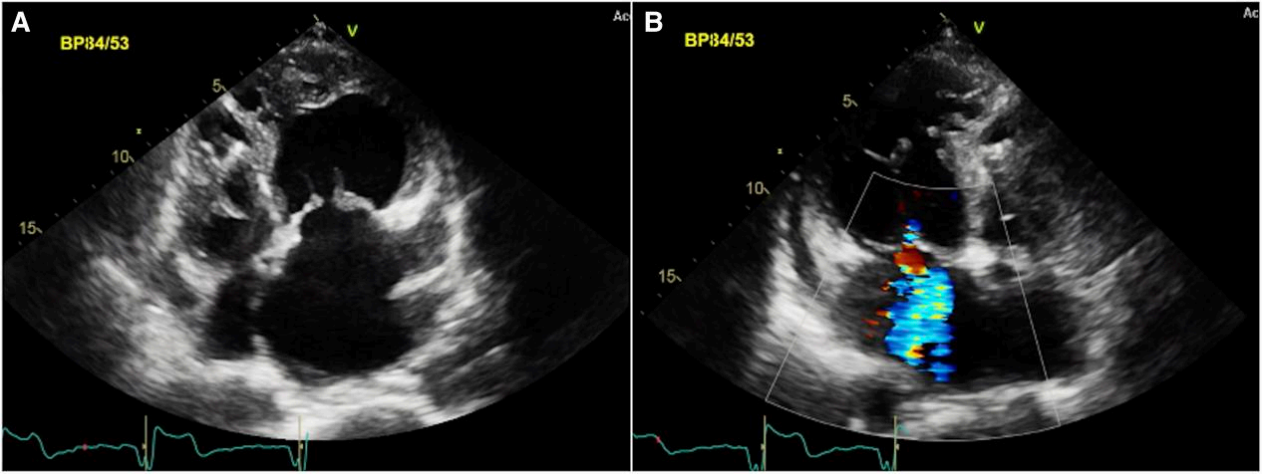
Transthoracic echocardiography of a 57-year-old male patient with ccTGA. *(A)*: apical four-chamber-view revealing the systemic RV with a hyper trabeculated apex in leftward position with accompanying moderate regurgitation of the systemic tricuspid atrioventricular valve in the apical two-chamber view *(B)*.

Repeat imaging revealed a dilated left atrium (LA volume index 113 mL/m^2^), a dilated left-sided sRV (end-diastolic diameter 63 mm, end-systolic diameter 54 mm) with reduced systolic function [sRV ejection fraction (EF) 26%], and moderate tricuspid regurgitation (TR) (*Figures 3A–D* and *4*). The estimated subpulmonic, morphologic LV systolic pressure was 53 mmHg. The cardiac rhythm was atrial fibrillation. The patient’s pacemaker was upgraded to a cardiac resynchronisation therapy device (CRT). Following CRT, no improvement occurred in RV EF, but the estimated morphologic LV systolic pressure decreased to 35 mmHg, with initial improvement in the patient’s symptoms. However, over the next few years, the patient’s symptoms gradually worsened, resulting in multiple hospitalisations for heart failure. Chest radiography showed significant cardiomegaly (*Figure 2B*) and serial follow-up echocardiograms documented a steady decline in sRV function, moderate TR, and elevated subpulmonary LV pressures (60–75 mmHg). Sacubitril/valsartan was added, and he has now been listed for heart transplantation. The patient is currently hospitalized on intravenous inotropes with active planning for a ventricular assist device (VAD) as a bridge to transplant.

## Case 4: acute coronary syndrome—an unusual presentation

A 63-year-old man, previously well, in NYHA FC I presented to his local hospital with acute onset chest pain associated with shortness of breath and ankle oedema. A diagnosis of an acute coronary syndrome (ACS) and heart failure was made. Cardiovascular risk factors included a 5-year history of hypertension, sub optimally managed diabetes, and a history of smoking. He had been asymptomatic until a few weeks prior to presentation.

Angiography revealed triple-vessel disease; however, unusual coronary anatomy resulted in referral to an ACHD centre. Dual antiplatelet therapy, metoprolol, spironolactone, atorvastatin, trimetazidine, metformin, and insulin had been commenced for his coronary artery disease and diabetes.

**Figure 5:**
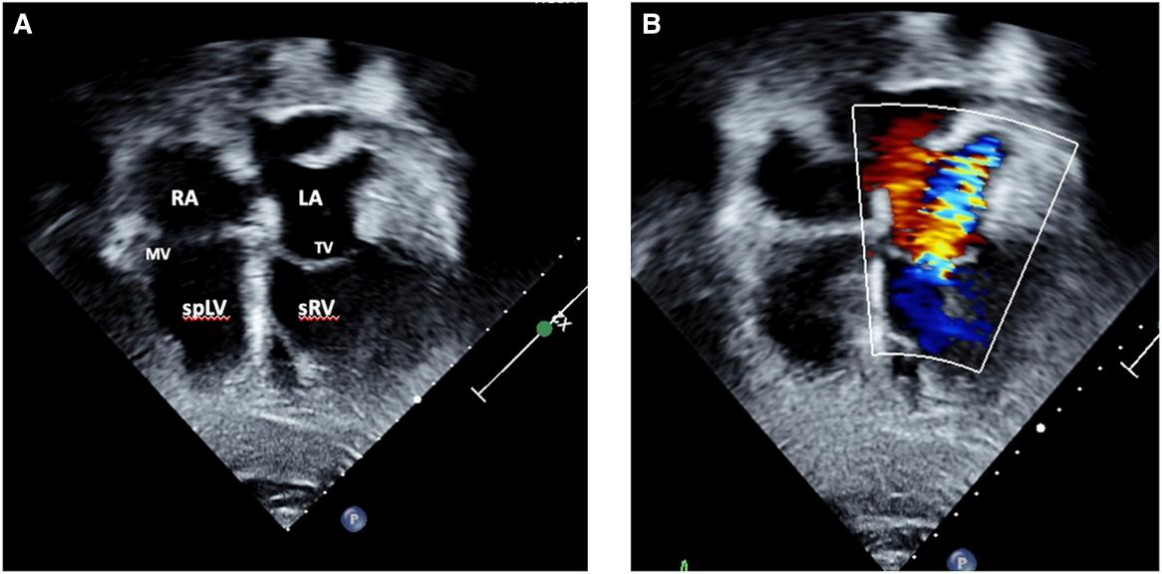
Transthoracic echocardiography of a 63-year-old male presenting with ACS and incidental findings of *(A)* atrio-ventricular and ventriculo-arterial discordance in the setting of ccTGA with pulmonary stenosis, VSD as well as moderate regurgitation of the systemic AV-valve *(B)*. LA, left atrium; MV, mitral valve; spLV, sub pulmonary left ventricle; sRV, systemic right ventricle, TV, tricuspid valve.

**Figure 6:**
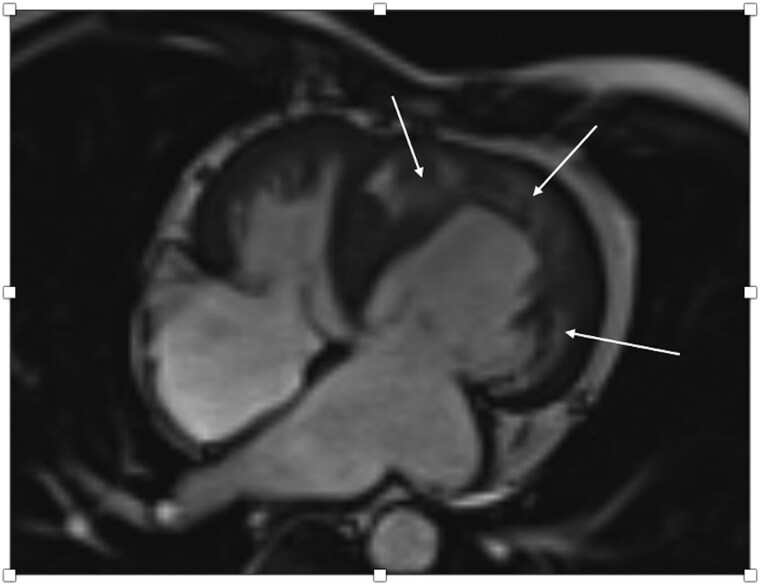
Cardiac MRI of a 63-year-old male with ACS after PCI showing impaired sRV function as well as subendocardial and transmural oedema in the sRV wall as well as the interventricular septum (arrows) correlating with an acute NSTEMI due to severe stenosis of the middle RCA and a CTO of both the left circumflex artery and the distal RCA.

Transthoracic echocardiography confirmed atrio-ventricular and ventriculo-arterial discordance as well as an unrestrictive VSD and severe PS (peak gradient 91 mmHg, mean 65 mmHg) (*Figure 5*). The systemic atrioventricular valve (tricuspid valve) was moderately regurgitant and sRV function impaired (tricuspid annular plane systolic excursion was 1 cm, RV fractional area change 25%, and tissue Doppler imaging’s 5 cm/s). Subpulmonary left ventricular function was preserved (*Figure 6*).

**Figure 7:**
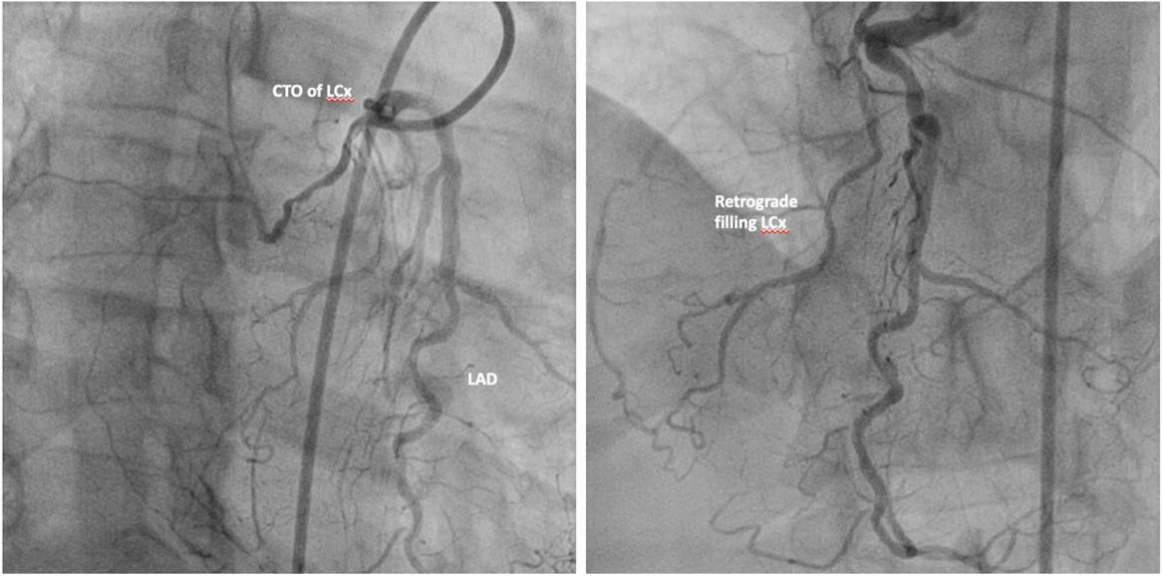
Coronary angiogram of a 63-year-old-male presenting with ACS. LCA from the right coronary cusp revealing CTO of the left circumflex artery with retrograde filling. CTO, chronic total occlusion; LAD, left anterior descending artery; LCx, left circumflex artery.

**Figure 8:**
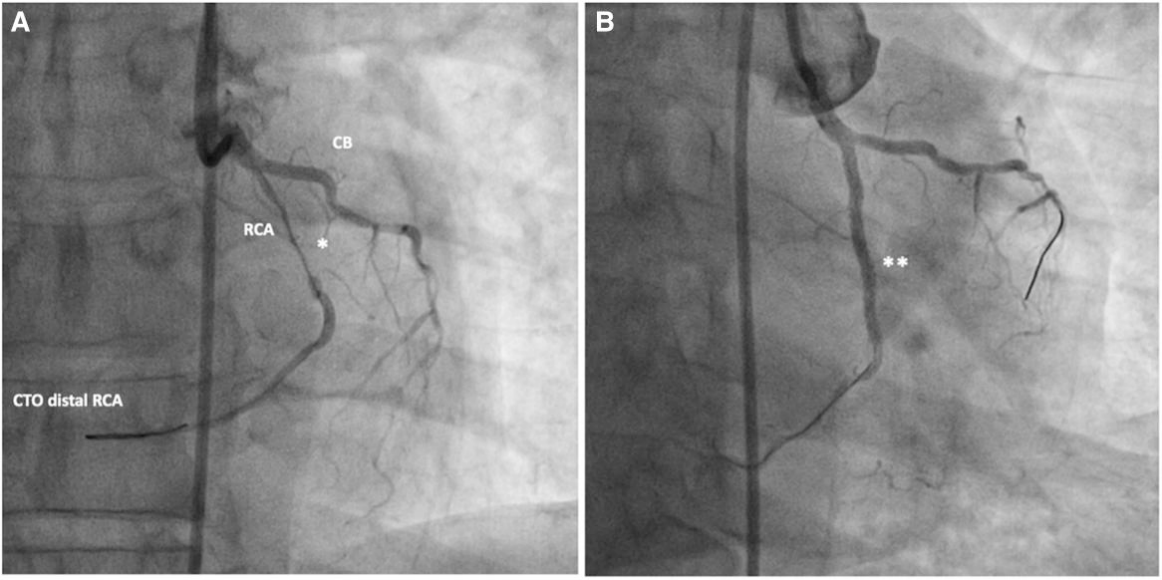
Coronary angiogram of a 63-year-old male presenting with ACS. RCA from the left coronary cusp revealing severe stenosis of the mid-RCA *(A)* and CTO of the distal RCA. *(B)* Post-interventional angiogram after PCI of the mid-RCA with a good interventional result with remaining CTO of the distal RCA after unsuccessful interventional attempts. CB, Conal branch; CTO, chronic total occlusion; *, severe stenosis of mid-RCA; **, post-stenting of mid-RCA.

The coronary angiogram (*Figures 7* and *8*) had revealed the inverse coronary anatomy of ccTGA with the right coronary artery (RCA) originating from the left coronary cusp and the left coronary from the right coronary cusp. There was chronic total occlusion (CTO) of the left circumflex artery with retrograde filling (*Figure 7*), mild disease of the left anterior descending artery, and severe stenosis of the middle RCA with distal CTO (*Figure 8A*). Percutaneous coronary angioplasty (PCI) and stenting of the RCA were performed (*Figure 8B*). Attempts to cross the CTO of the circumflex and distal RCA were unsuccessful.

He was reviewed by a multi-disciplinary team. PCI improved his heart failure symptoms and functional capacity. His NT pro BNP dropped from 1771 to 737 pg/mL and he remains angina-free upon recent clinical examination follow-up.

## Discussion

The cases presented highlight the heterogenous presentation and complications of unoperated ccTGA throughout the life span. They showcase different aspects of the disease and emphasize that interdisciplinary management and ACHD centre expertise are key to optimal care.^5^

The life expectancy of patients with ccTGA is reduced with 50% being alive at the age of 40 in patients with associated lesions and 50% being alive at the age of 60 in patients without associated lesions,^1^ In balanced ccTGA patients often remain asymptomatic into adulthood. First symptoms arise from arrhythmias, AV conduction abnormalities, worsening sRV function, and insufficiency of the systemic (tricuspid) AV-valve.

The key to managing ccTGA is recognising the morphology on multimodality imaging,^1,6^ including echocardiography, cardiac computed tomography (CCT) and CMR.^7^ This should be followed by serial right heart catheterisation (RHC), CPET^8^ and evaluation of cardiac biomarkers as clinically indicated.^9,10^ Though TTE is the primary modality,^2^ CMR is the gold standard to assess sRV function and anatomy.^1^ Clues on echocardiography are the parallel course of the aorta and the pulmonary artery, the trabeculated endocardial surface of the sRV, the moderator band, and the apically displaced, sometimes Ebsteinoid systemic tricuspid AV valve.^1–3^ Complementary imaging modalities, such as CCT, TOE, and 3D-imaging, may further support the diagnosis.

Most recommendations for diagnosis and management are based on consensus-level evidence. The focus should be on early treatment initiation for arrhythmias and heart failure, as this may prevent or delay further deterioration and disease progression.^11–14^

Sudden cardiac arrest may be the first manifestation. Current guidelines recommend secondary preventive ICD implantation when reversible causes can be excluded.^1^ Electrophysiological evaluation and consideration of ablation in specialized ACHD centres is recommended as first line management.^1^ Anti-arrhythmic drugs are utilised in the setting of recurrent arrhythmias with appropriate attention to monitoring of side-effects as relevant.

Primary prevention ICD evidence-based data are lacking with the mechanism and implications of sRV function deterioration not fully understood.

Complete heart block and AV conduction abnormalities due to the anterior left position of the His bundle (AV conduction is said to decline at a rate of 2%/year^1^) are common in ccTGA with increasing indication for permanent pacemaker (PPM) implantation. If the pacing index is high (>40%) upgrade to CRT is recommended,^1^ although it is unclear whether an early upgrade can preserve biventricular function.^14^

To date, there are no specific guideline recommendations on HF medication for worsening sRV function. ACEi or ARB, beta-blockers and diuretics are used. Sacubitril/Valsartan has been shown to be beneficial in the setting of sRV failure.^15^ The effect of SGLT-2i remains to be determined. Progressive systemic AV valve regurgitation propagates haemodynamic deterioration. The timing of AV valve replacement is critical and should be guided by the severity of regurgitation, sRV function, and patient symptoms. RHC, CPET^16^ and serial NT-proBNP may provide additional information to guide therapy.

In end-stage heart failure,^17,18^ heart transplantation should be considered, but organ allocation can be difficult, resulting in deterioration or death on the waiting list. In this setting, VAD implantation may serve as a bridge to transplant or as destination therapy. VAD implantation in ccTGA has technical difficulties, especially with inflow cannulation to the predominantly trabeculated anatomical RV with a need for anastomosis to the anterior RV wall.^19^

Pregnancy carries a risk of worsening heart failure given the limited haemodynamic adaptive capacity of the sRV. Current guidelines recommend pre-conception counselling with assessment of maternal risk (utilising the mWHO classification) and regular review at a specialized centre with a cardio-obstetric unit.^6^

Patients with ccTGA may additionally present with acquired cardiovascular events later in life^17^ and abnormal anatomy of the coronary arteries may be the first indicator of their diagnosis.

The objective of this case series is to enhance the comprehension of the application of contemporary guidelines and evidence-based methodologies in the management of ccTGA. By illustrating the spectrum of clinical scenarios and management strategies, it is hoped to provide a practical resource for clinicians navigating the complexities of this rare condition.

## Data Availability

The data underlying this article will be shared on reasonable request to the corresponding author.

## Lead author biography

Maria Luisa Benesch Vidal is a junior cardiologist currently in training at the University Heart and Vascular Centre in Hamburg, Germany. She completed her medical studies at the University of Münster, Germany, where she also completed her doctoral thesis on the use of artificial intelligence in the diagnosis and prognostic assessment of PAH. Her investigatory fields of interest are adult congenital heart disease and heart failure.
